# Corrigendum to “Comparison of baseline outcomes between surgical and nonoperative management in youth with lower extremity torsional abnormalities” [J Orthopedics 73 (2026) 227–233]

**DOI:** 10.1016/j.jor.2026.02.029

**Published:** 2026-02-06

**Authors:** M. Gagnon, J.P. Bauer, K.M. Kruger, S. Tavukcu, H. Altiok, R. Hamdy, M. Bernstein, L.N. Veilleux

**Affiliations:** a Department of Surgery, McGill University, Montreal, QC, Canada; b Shriners Hospitals for Children-Canada, Montreal, QC, Canada; c Shriners Children's-Portland, Portland, OR, USA; d Shriners Children's-Chicago, Chicago, IL, USA; e Marquette University, Milwaukee, WI, USA

**Keywords:** Lower extremity torsional abnormality, Femoral anteversion, Tibial torsion, Derotational osteotomy, Nonoperative management, Pediatric orthopaedic

## Abstract

**Introduction:**

Lower extremity torsional abnormalities (LETA) include excessive femoral and/or tibial torsion and can be associated with pain, functional limitations and patellofemoral instability. This study examined whether clinical differences exist between surgically and nonoperatively managed patients.

**Methods:**

Patients aged 10-21 years with LETA referred for nonoperative or surgical treatment underwent imaging, physical assessment (range of motion (ROM), handheld dynamometry, patellofemoral tests), gait analysis and patient-reported outcomes (PROs: PODCI, FAQ, PEDI-IKDC, APPT, Cosmetic ratings, PROMIS, HAGOS). A control group of typically developing participants underwent the same assessments except imaging. Groups were compared using ANOVA/Kruskal-Wallis test, and statistical parametric mapping.

**Results:**

Thirty-four patients (surgery n = 21, 16.3 ± 2.3 years; nonoperative n = 13, 14.7 ± 2.0 years) and 21 controls (15.8 ± 2.7 years) were included. Femoral version, tibial torsion, ROM, and muscle strength did not differ between treatment groups. Signs of patellofemoral instability were more frequent in the surgical group (MPFL laxity: 14/21 vs 1/13, p < 0.001; apprehension: 13/19 vs 1/13, p < 0.001; J-sign: 11/21 vs 1/13, p = 0.01). Pain was similar between treatment groups (PODCI Pain ≈58%; APPT ≈3–4/10; PROMIS Pain Interference 53–54). PROs showed a tendency toward worse knee function in the surgical group (PEDI-IKDC: 55.0 ± 18.0 vs 67.5 ± 14.6), while the nonoperative group scored lower on HAGOS Activity of Daily Living (96.0 ± 10.2 vs 81.9 ± 22.4, p = 0.01) and Function in Sport and Recreation (87.3 ± 20.2 vs 73.8 ± 24.3, p = 0.03). Gait differences were limited. The nonoperative group demonstrated greater knee flexion at terminal stance than controls.

**Conclusion:**

Both surgical and nonoperative patients differed from age-matched controls across multiple measures, supporting the need for treatment in either pathway. However, differences between the treatment groups were modest aside from greater patellofemoral instability in the surgical group. These findings highlight the need for clear, consensus-based guidelines to define treatment pathways.

The authors regret that the abstract was omitted from the published version of this article. The abstract is provided below.

The authors would like to apologise for any inconvenience caused.

